# Effect of high salinity and of priming of non-germinated seeds by UV-C light on photosynthesis of lettuce plants grown in a controlled soilless system

**DOI:** 10.3389/fpls.2023.1198685

**Published:** 2023-07-04

**Authors:** Salah Fgaier, Jawad Aarrouf, Félicie Lopez-Lauri, Yves Lizzi, Florine Poiroux, Laurent Urban

**Affiliations:** ^1^ Unité Propre de Recherche Innovante, Equipe de Recherche et d'Innovations Thématiques (ERIT) Plant Science, Interactions and Innovation, Avignon Université, Avignon, France; ^2^ Nova Genetic, Zone Anjou Actiparc de Jumelles, Longué-Jumelles, France

**Keywords:** chlorophyll fluorescence, growth, leaf gas exchange, lettuce, salinity stress, seed priming, phytohormone balance, UV-C

## Abstract

High salinity results in a decrease in plant photosynthesis and crop productivity. The aim of the present study was to evaluate the effect of UV-C priming treatments of lettuce seeds on photosynthesis of plants grown at high salinity. Non-primed and primed seeds were grown in an hydroponic system, with a standard nutrient solution, either supplemented with 100 mM NaCl (high salinity), or not (control). Considering that leaf and root K^+^ concentrations remained constant and that chlorophyll fluorescence parameters and root growth were not affected negatively in the high salinity treatment, we conclude that the latter was at the origin of a moderate stress only. A substantial decrease in leaf net photosynthetic assimilation (A_net_) was however observed as a consequence of stomatal and non-stomatal limitations in the high salinity treatment. This decrease in A_net_ translated into a decrease in growth parameters; it may be attributed partially to the high salinity-associated increase in leaf concentration in abscisic acid and decrease in stomatal conductance. Priming by UV-C light resulted in an increase in total photosynthetic electron transport rate and A_net_ in the leaves of plants grown at high salinity. The increase of the latter translated into a moderate increase in growth parameters. It is hypothesized that the positive effect of UV-C priming on A_net_ and growth of the aerial part of lettuce plants grown at high salinity, is mainly due to its stimulating effect on leaf concentration in salicylic acid. Even though leaf cytokinins’ concentration was higher in plants from primed seeds, maintenance of the cytokinins-to-abscisic acid ratio also supports the idea that UV-C priming resulted in protection of plants exposed to high salinity.

## 1 Introduction

The world’s population is expected to reach 9.8 billion in 2050 (FAO, 1999). An increase in food production is thus essential to feed this increasing population. Food security depends on several factors, including abundance and quality of water resources. Salinity is considered as one of the main environmental stresses that reduces agricultural productivity around the world (Läuchli and Grattan, 2007). Twenty percent (45 million ha) of the current 230 million ha of irrigated land is already salinized. Furthermore, the area of saline soil is expected to increase worldwide, reaching 50% in 2050 (FAO, 2008).

Salt stress-related effects on agricultural yield are extremely harmful. Under salinity, crops exhibit slower growth rates and significantly reduced reproductive development (Munns and Tester, 2008; Roy et al., 2014). The negative salinity effects are first related to osmotic stress followed by salt toxicity (Munns, 2002), but also impaired nutritional balance and oxidative stress (Chaves et al., 2009; Isayenkov and Maathuis, 2019; Rasheed et al., 2020). Osmotic stress causes a rapid inhibition of the rate of expansion of young leaves and a significant decrease in stomatal conductance of older leaves (Munns and Tester, 2008). Salt toxicity occurs over time as very high Na^+^ concentrations accumulate within plants. Toxic concentrations of salt inhibit the synthesis of proteins and metabolites that are essential for plant growth. These perturbations affect the carbon balance mainly through the reduction of photosynthesis (Ashraf and Harris, 2013), as well as sugar metabolism, phloem loading, sucrose translocation from source to sink organs (Pattanagul and Thitisaksakul, 2008; Lemoine et al., 2013; Penella et al., 2016), resulting in reduced plant growth and yield. In the chloroplast, salt stress induces the production of ROS, which can be deleterious if the antioxidant mechanisms are not able to eliminate them. The generated ROS not only induce photoinhibition by disrupting electron flow, but also affect PSII repair mechanism. ROS may notably inhibit the synthesis of protein D1 (Nath et al., 2013; Gururani et al., 2015).

Substantial changes in hormone concentrations and balance are associated with salinity stress (Fahad et al., 2015; Yu et al., 2020). These changes are behind the negative effects of high salinity on photosynthesis and other functions related to growth and development, but the same changes are also involved in adaptative processes aiming at increasing tolerance against high salinity. The view can be reversed. Changes in hormone concentrations and balance result from the triggering of adaptative processes, which may also result in decreased photosynthesis and growth. For instance, rapid production of abscisic acid (ABA) is essential for plant survival in conditions of drought and high salinity because of its role as a long-distance signal controlling stomatal conductance (g_s_); xylem ABA concentration indeed controls g_s_ (Zhang et al., 2006), therefore preventing excessive losses of water that threaten plant survival in conditions of drought or high salinity, i.e. of restricted water availability. But substantial decreases in g_s_ come at the price of reduced net photosynthetic assimilation rate (A_net_). Reduced A_net_ not only translates into reduced growth but also increased risk of photooxidative stress, which is associated to excess of energy absorbed by photosystems under the form of photons relative to the quantity of energy used by photochemistry, and the resulting production of reactive oxygen species (ROS) by the photosynthetic machinery. ABA itself regulates, along with other hormones (auxin, salicylic acid, cytokinins), ROS scavenging processes (Chaves et al., 2009). Auxin mediates numerous responses to stress *via* a nuclear pathway that can activate or repress several genes by recruiting specific DNA-binding transcription factors called ARFs (auxin response factors) (Roosjen et al., 2018; Verma et al., 2022). Downregulation of ARF4 in tomato results in increased root growth and leaf chlorophyll content, and upregulation of CAT1 (catalase 1), Cu/ZnSOD (superoxide dismutase), MDHAR (monodehydroascorbate reductase) in conditions of drought (Bouzroud et al., 2020). ABA and auxin pathways interact antagonistically in plant tolerance mechanisms. Chen et al. (2021) showed that ARF4 knockout resulted in upregulated ABA signaling by activation of SCL3 (SCARECROW-LIKE 3) transcription factor. The antagonist interaction between ABA and auxin is attested in a spectacular way by the observation that auxin can promote stomatal opening and reverse stomatal closure induced by ABA (Levitt et al., 1987).

Salicylic acid (SA) occupies a prominent place among plant hormones thanks to its pivotal role in both resistance against biotrophic and semi-biotrophic pathogens and tolerance against abiotic stress (Urban et al., 2022). SA receptor, NPR1 (non-expresser of pathogenesis-related protein 1), is indeed believed to be the master regulatory protein of defense and tolerance responses by being a transcriptional co-activator not only of PR genes (Jayakannan et al., 2015), but also of genes of tolerance against abiotic stress (Iglesias et al., 2022). The role of SA signaling and NPR1 is believed essential for controlling Na^+^ entry into roots and K^+^ accumulation in shoots in conditions of high salinity (Rasheed et al., 2020). SA not only exerts a positive effect on photosynthesis and growth in stressed plants (Urban et al., 2022), it also stimulates antioxidant activity, as shown on salt-stressed *Triticum aestivum* (Li et al., 2012) and tomato (Csiszár et al., 2014) plants. See also the review of Rasheed et al. (2020).

Cytokinins (CKs) have recognized roles in plant growth and development, senescence delay, as well as tolerance to biotic and abiotic stress (Kieber and Schaller, 2018; Cortleven et al., 2019). Stress conditions may lead to an increase in CKs concentrations in plants, as it was observed in maize and tobacco (Alvarez et al., 2008; Dobra et al., 2010). Nishiyama et al. (2011) observed that high salinity triggers up-regulation of ADENOSINE ISOPENTENYL TRANSFERASES (IPTs), which are key genes for CKs synthesis. Overproduction of endogenous CKs levels enhances drought stress tolerance in many plants but reduced levels also have been found to exert positive effects (Mandal et al., 2022). Generally speaking, the role in CKs in stress tolerance must be thought in conjunction with the other hormones. Considering salinity stress, CKs enhance the expression of genes involved in antioxidant activities (Arif et al., 2020), but they also activate genes involved in the type-A response, which suppresses expression of ABSCISIC ACID INSENSITIVE 1 (AB15). It ultimately leads to the downregulation of genes involved in ABA signaling. Genes like CYTOKININ RESPONSE 1 (CRE-1) and transcription factor ARR-B are downregulated under salinity (Rao et al., 2016; Wang et al., 2020). CKs could eliminate tolerance to high salinity in plants by interacting antagonistically with ABA (Javid et al., 2011). Conversely, accumulation of stress-induced ABA downregulates CKs production *via* MYB2 transcription factor (Li et al., 2016). By contrast, CKs are credited with a positive interaction with SA (Jiang et al., 2013).

Improving the ability of plants to maintain growth and productivity in saline soils represents a major challenge to ensure food safety in the future. Possible ways and techniques to increase salinity tolerance have been the subject of several studies. The most common technique used for this purpose is genetic methods (conventional breeding programs, genetic engineering, interspecific hybridization, etc.). However, the genetic complexity of this trait reduces the efficiency of this technique. Consequently, the successful use of this technique to produce a salt-tolerant cultivar is very low (Flowers, 2004; Munns, 2005; Jisha et al., 2013; Cabello et al., 2014). Clearly there is the need to find ways to increase salt tolerance by non-genetic approaches, such as seed priming (Jisha et al., 2013).

Priming is defined as “a physiological process by which a plant prepares to respond to imminent abiotic stress more quickly or aggressively” (Thomas and Puthur, 2017; Dutta, 2018). Seed priming is a presowing approach for influencing seedling development by stimulating pregermination metabolic activities prior to the emergence of radicles and thus improving the germination rate, performance of the plants, and the plant tolerance to biotic and abiotic stresses (Afzal et al., 2016; Araújo et al., 2016). The enhanced tolerance of plants to abiotic stress by priming can be attributed to the activation of different stress-related metabolic processes (Thomas and Puthur, 2017).

Conventional seed priming consists of partially hydrating the seed to a point where germination processes are begun but not completed (Bilalis et al., 2012; Farooq et al., 2019). To enhance the growth and yield of many crop species under salinity stress, many seed priming agents have been reported in different articles, notably hydropriming (Srivastava et al., 2010; Amooaghaie, 2011), osmopriming (Sivritepe et al., 2005; Ahmed et al., 2017), nutriopriming (Harris et al., 2007; Harris et al., 2008; Khan et al., 2019; Moatter, 2020), and hormonal priming (Zhang et al., 2007; Afzal et al., 2011; Afzal et al., 2013). These conventional methods are accompanied by several drawbacks, such as a long treatment duration, high labor costs and, in the case of hormonal priming, negative environmental impacts. It is therefore necessary to develop fast, effective and more environmentally friendly priming protocols to replace these conventional techniques (Araújo et al., 2016; Dutta, 2018; Farooq et al., 2019).

Physical treatment can be considered as an alternative to conventional methods (Afzal et al., 2016). Among physical treatments, seed priming by UV-C light arouses increasing interest. UV-C radiation was initially explored for its germicidal effects, mainly in the food industry and for its potential for improving the post-harvest physiology of fruits and vegetables (Jagadeesh et al., 2011; Bravo et al., 2012). Recently, it has been observed that low doses of UV-C light (hormetic doses), applied to growing plants, can stimulate plant defenses against fungal diseases (Ouhibi, 2015; Urban et al., 2016; Vàsquez et al., 2017; Aarrouf and Urban, 2020; Forges et al., 2020; Vàsquez et al., 2020). UV-C seed priming effects have been reported by several researchers who have clearly demonstrated its positive impact on germination, seedling growth, and final yield. Additionally, UV-C seed priming can increase plant disease resistance. Cabbage seed treatment with UV-C light reduces the incidence of *Xanthomonas campestris* pv. campestris on plants by 75% (Brown et al., 2001). Siddiqui et al. (2011) observed reductions in disease development caused by Fusarium spp., *Rhizoctonia solani* and *Macrophomina phaseolina* on mung bean and groundnut plants. Recently, Scott et al. (2019) reported reductions in the incidence and progression of *Botrytis cinerea* and *Fusarium oxysporum* in tomato plants.

However, to our knowledge, there is only one study about dry seed treatment with UV-C light (Ouhibi et al., 2014). The latter observed a strong positive effect in lettuce plants grown at high salinity. They moreover observed that the positive effect of seed priming by UV-C light was associated with an increase in leaf antioxidant activity and in the content of phenolic compounds, but they did not investigate the way growth components were affected, notably leaf net photosynthetic assimilation (A_net_), nor did they investigate the possible role of a change in the hormonal balance.

The major objective of the present study was to explore the effects of UV-C seed priming on photosynthesis of lettuce plants grown at high salinity. Special care was taken to control NaCl concentration in the nutrient solution. In addition to photosynthesis and growth parameters, we investigated the effects of UV-C seed priming on leaf concentrations in SA, ABA, auxin and CKs.

## 2 Materials and methods

### 2.1 Seed priming with UV-C radiation

Lettuce seeds (*Lactuca sativa* L. cv. Eden), provided by Nova Genetic (Longué-Jumelle, France), were treated using UV-C amalgam lamps at 254 nm supplying 50 mW cm^-2^ (UV Boosting, Boulogne-Billancourt, France). UV-C intensity was measured using a radiometer (RM-12. Opsytec Dr, Groebel. Germany) fitted with a 254 nm sensor. Preliminary experiments were carried out to test the effects of many UV-C doses ranging from 1 to 1000 kJ/m^2^ on plant growth. Results of these experiments (not shown here) allowed to identify the most effective dose of UV-C radiation: 200 kJ m^-2^. Seeds were exposed to UV-C treatment for 6 minutes 40 seconds. Pr and NPr abbreviations are used to refer to lettuce plants from primed seeds with 200 kJ m^-2^ and control plants issued from nonprimed seeds, respectively.

### 2.2 Growth conditions and experimental design

**Figure 1:**
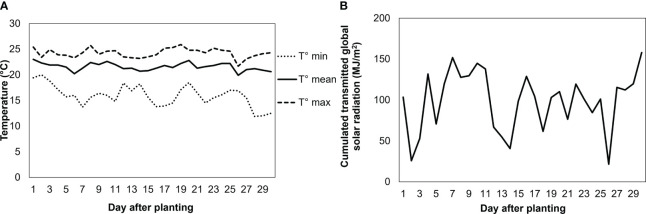
Daily mean, maximum, and minimum temperatures **(A)**, and daily cumulated transmitted global solar radiation at experiment **(B)**. Greenhouse of Avignon university (France) 43°54’43.3”N 4°53’21.0”E.

**Figure 2:**
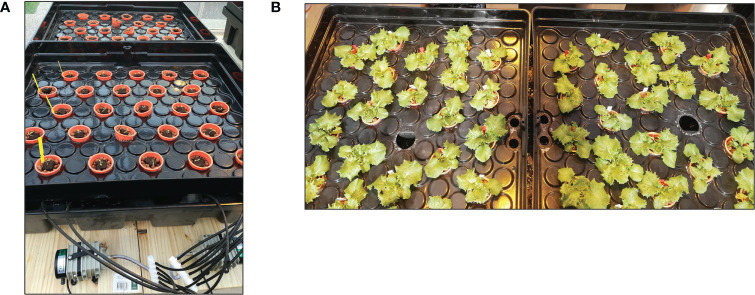
The hydroponic system at the time of planting **(A)**, and lettuce plants one week after planting **(B)**.

The experiment was performed in the greenhouse of Avignon University (France). Mean air temperature was 22.1°C (min: 11.9°C; max: 25.9°C) (**Figure 1**), and mean relative humidity 28% during the day and 78% during the night. Photosynthetically active radiation (PAR) of 450 ± 50 μmol photons m^-2^s^-1^ was provided by high-pressure sodium (HPS) lamps (400 Watts, Hortilux Schréder b.v., The Netherlands). Primed and nonprimed seeds were directly sown in rockwool cubes, where the seedlings were grown. Three weeks later, 96 plants were transferred to individual pots (7 cm in diameter) filled with clay pebbles and placed in a randomized block design on a hydroponic system specially designed to avoid the build-up of ionic gradients while maintaining a high level of oxygenation in the root environment (**Figure 2**). The system consisted of two plastic tanks (2 m^2^) located next to each other, filled with the nutrient solution and without NaCl for the first one, and the same nutrient solution plus 100 mM NaCl for the second one, the so-called high salinity treatment. The hydroponic nutrient solution was comprised of 7% N, 4% P_2_O_5_, 5% K_2_O, 0.036% Fe, 0.01% Mn, and 0.004% Zn. The electrical conductivity (EC) of the nutrient solution was set at 2.6 ± 0.3 dS m^−1^ in the control tank. In the presence of NaCl solution, EC reached ca. 10 ± 1 dS m^−1^. NaCl was supplied at the time the plants were transferred to the plastic tank. In each tank, an air pump (32 W, >0.02 MPa, 60 l min^-1^; HAILEA, China) with 6 immersed outlets was operated continuously to keep the concentrations of the minerals homogeneous in the nutrient solution and to supply oxygen to the roots.

The nutrient solution was completely changed every 10 days. pH was monitored daily and maintained at 6.0 ± 0.4 using a portable pH/EC/temperature meter (HANNA instruments, Portugal).

Prior to the onset of the trial, light and temperature measurements were performed at different times of the day to check that there were no climate differences between the two tanks. UV-C-treated (Pr) and control plants (NPr) were randomly distributed in each tank (control and with NaCl).

### 2.3 Growth and yield parameters

Twenty-one days after planting in the hydroponic system, the plants were harvested. The fresh mass of the leaves and roots was measured. For dry mass determination, the plant material was kept at 80°C in an oven for 48 h. Individual and total leaf areas were determined using ImageJ® (https://imagej.nih.gov/ij/) prior to the dry mass measurement.

### 2.4 Determination of Na^+^ and K^+^ concentrations

Concentrations of Na^+^ and K^+^ were determined in the dry plant material and expressed on a dry mass basis. Roots were rinsed three times in cold distilled water after harvest. Cation extraction was realized with HNO_3_ (0.5%) and estimated by standard flame photometry (PFP7, JENWAY, UK).

### 2.5 Leaf gas exchange measurements

Seven and 14 days after planting in the hydroponics system, leaf gas exchange measurements were carried out with an infrared CO_2_/H_2_O gas analyzer and leaf chamber system with an external light source in the 400-700 nm range (LI 6800, Licor, Lincoln, USA). The net CO_2_ assimilation rate (A_net_) and leaf conductance of water vapor (g_s_) were measured between 10 a.m. on young, fully expanded leaves, with a level of PAR set at 400 μmol photons m^-2^s^-1^ and a partial pressure of ambient CO_2_ (Ca) at 40 Pa. At the end of the A_net_ measurements, the light source was turned off (PAR = 0 μmol photons m^-2^ s^-1^) for 3 min, and the dark respiration rate (R_d_) was measured.

**Figure 3:**
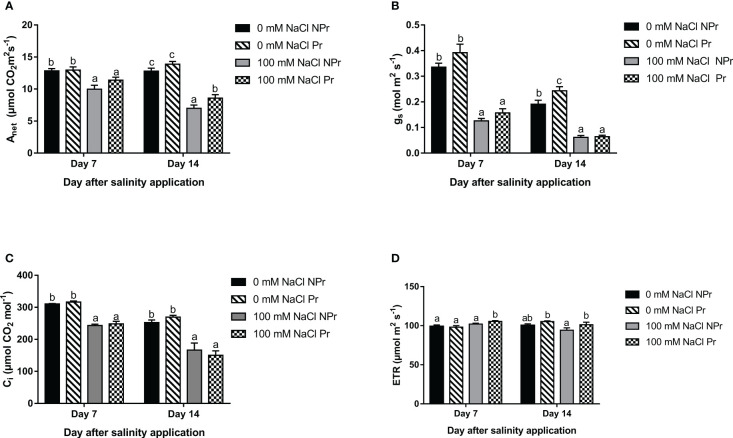
Effects of lettuce seed priming with UV-C radiation on leaf net photosynthesis of plants grown in high salinity conditions (100 mM NaCI) or in the absence of NaCI (0 mM). Plants derived from seeds primed are designed by Pr, and non-primed by NPr. A_net_ represents net carbon assimilation rate **(A)**, g_s_ stomatal conductance **(B)**, Ci intercellular CO_2_ concentration **(C)** and ETR electron transport rate **(D)**. Error bars represent the standard errors (n=10). Different letters indicate significant differences among means for each measurement date after salinity application at p<0.05 according to Dunn’s test.

Photosynthetic capacity is commonly assessed through measurements of V_cmax_, the maximum carboxylation rate of Rubisco, and J_max_, the light-saturated electron transport rate. Their values can be derived from so-called A-C_i_ curves (Urban et al., 2017). Low g_s_ at day 7, and even more at day 14 (**Figure 3B**) prevented us to obtain reliable A-C_i_ curves. The photosynthetic capacity was therefore simply estimated as A_max_, the maximal rate of net photosynthesis under saturating conditions. PAR was set at 2000 μmol photons m^-2^ s^-1^ and Ca at 2000 Pa. A_max_ was measured 7 and 14 days after transplanting the plants.

### 2.6 SPAD values

Seven and 14 days after planting, the leaf chlorophyll content was estimated by taking the mean of three readings with a portable Chlorophyll meter (Minolta Co. Ltd, Osaka, Japan).

### 2.7 Chlorophyll fluorescence measurements and an analysis of fluorescence transients using ChF induction curves

On the same day as the leaf gas exchange measurements (7 and 14 days after salinity application), chlorophyll fluorescence (ChlF) transients were measured before 10 a.m. with a portable Pocket PEA chlorophyll fluorimeter (Hansatech Instruments, King’s Lynn, Norfolk, United Kingdom). Leaves were dark adapted for 20 min with a lightweight plastic leaf clip prior to measurement and then exposed for 1 s to 3500 μmol photons m^-2^ s^-1^ (637 nm peak wavelength). The chlorophyll fluorescence intensity at t = 50 μs was considered F_0_ (Strasser and Strasser, 1995). The fast ChlF kinetics (from F_0_ to F_m_, where F_0_ and F_m_ are, respectively, the minimum and maximum measured ChlF of photosystem II (PSII) in the dark-adapted state) were recorded from 10 μs to 1 s. The ratio of variable ChlF (F_v_) to F_m_ (F_v_/F_m_), i.e. the maximum quantum yield of PSII (φP_0_ = TR_0_/ABS), the performance index (PI), a plant vitality indicator and its components (F_v_/F_0_, the variable to minimum ChlF ratio; RC/ABS, which represents the density of reaction centers expressed on an absorbed photon flux basis; (1–V_j_)/V_j_), where V_j_ is the relative variable ChlF at t = 2 ms) were calculated automatically (Strasser et al., 2000; Campa et al., 2017). We also calculated the dissipated energy flux on an absorbed photon flux basis (DI_0_/ABS), an indicator of the importance of processes other than trapping, and the electron transport fluxes from Q_A_ to Q_B_ (ET_0_) and from Q_B_ to PSI acceptors (RE_0_), expressed as quantum yields (φE_0_ = ET_0_/ABS and φR_0_ = R_0_/ABS). The latter parameter is arguably related to cyclic electron transport (CET) activity (Ripoll et al., 2016). Changes in CET activity play a major role in plant adaptations to stress. Moreover, we calculated the following parameters, which are indicators of potential damage: F_0_, F_v_/F_m_, V_k_/V_j_, and S_m_ (Ripoll et al., 2016; Urban et al., 2017). V_k_/V_j_ represents the ratio of variable ChlF at 300 μs (K-step) to variable ChlF at 2 ms (J-step), and S_m_ is the normalized area above the ChlF induction curve.

### 2.8 Determination of endogenous concentrations of phytohormones

Harvested lettuce leaves were immediately frozen in liquid nitrogen before being lyophilized. Endogenous phytohormone (abscisic acid, auxin, salicylic acid, and cytokinin) concentrations were determined by UPLC-XEVO in the Chemistry and Metabolomics platform of Jean-Pierre Bourgin Institute, INRAE Versailles (Grignon, France) following the method of Chauffour et al. (2019).

### 2.9 Statistical analysis

A randomized experimental design was used in this study. All data were analyzed using the Kruskal–Wallis nonparametric test and Dunn’s *post hoc* test with Bonferroni correction. Analyzed data correspond to the mean values (± standard error) of growth parameters (n=24), SPAD (n=24), water content (n=24), Na^+^ and K^+^ contents (n=10), photosynthetic components (n=10), fluorescence parameters (n=48), WUE (n=10), and phytohormone content (n=6). All statistical analyses were performed using R software.

## 3 Results

### 3.1 Effects of high salinity and UV-C seed priming on growth

**Figure 4:**
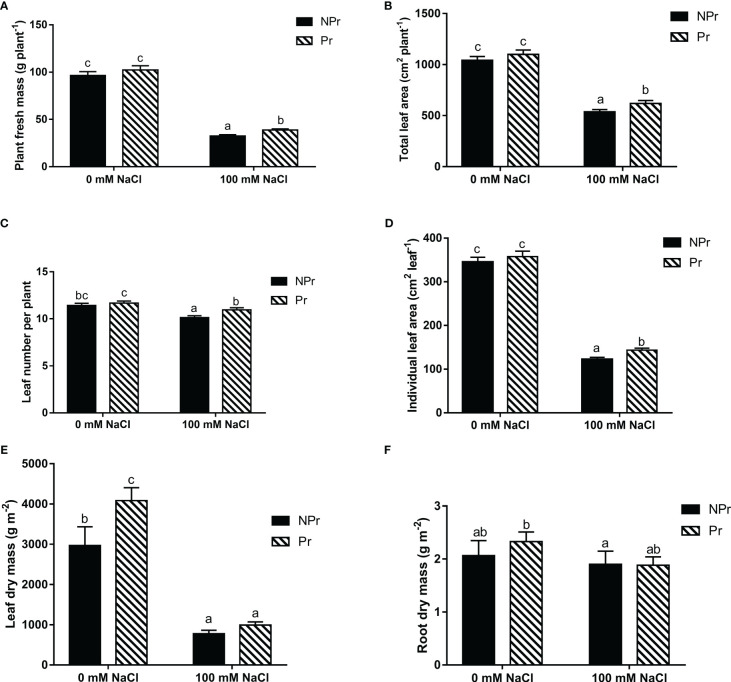
Effects of lettuce seed priming with UV-C radiation on growth and yield parameters of plants grown in high salinity conditions (100 mM NaCI) or in the absence of NaCI(0 mM). Plants derived from seeds primed are designed by Pr,and non-primed by NPr. **(A)** Plant fresh mass (g plant^-1^), **(B)** total leaf area per plant, **(C)** leaf number per plant, **(D)** individual leaf area, **(E)** leaf dry mass, **(F)** root dry mass.Error bars represent the standard errors (n=24). Different letters indicate significant differences at p<0.05 according to Dunn’s test.

Growth parameters for plants derived from UV-C primed (Pr) and nonprimed (NPr) seeds under control (0 mM NaCl) and salinity (100 mM NaCl) conditions are presented in **Figure 4**.

Salinity significantly reduced all plant growth parameters, with the exception of the root dry mass. Total plant fresh mass (yield), plant leaf number, total leaf area, average individual leaf area, and leaf dry mass were reduced by 66% (**Figure 4A**), 48% (**Figure 4B**), 11% (**Figure 4C**), 65% (**Figure 4D**), and 74% (**Figure 4E**), respectively, in the NaCl-treated plants compared with the control.

Seed priming also resulted in an increase in leaf dry mass of 38% in the plants grown without NaCl.

In the plants grown at high salinity, UV-C priming resulted in an increase in yield (+20%, **Figure 4A**), leaf number of the plants (+8%, **Figure 4B**), total leaf area (+15%, **Figure 4C**) and individual leaf area (+17%, **Figure 4D**) compared to unprimed seeds.

### 3.2 Effects of high salinity and UV-C seed priming on plant sodium and potassium contents

**Figure 5:**
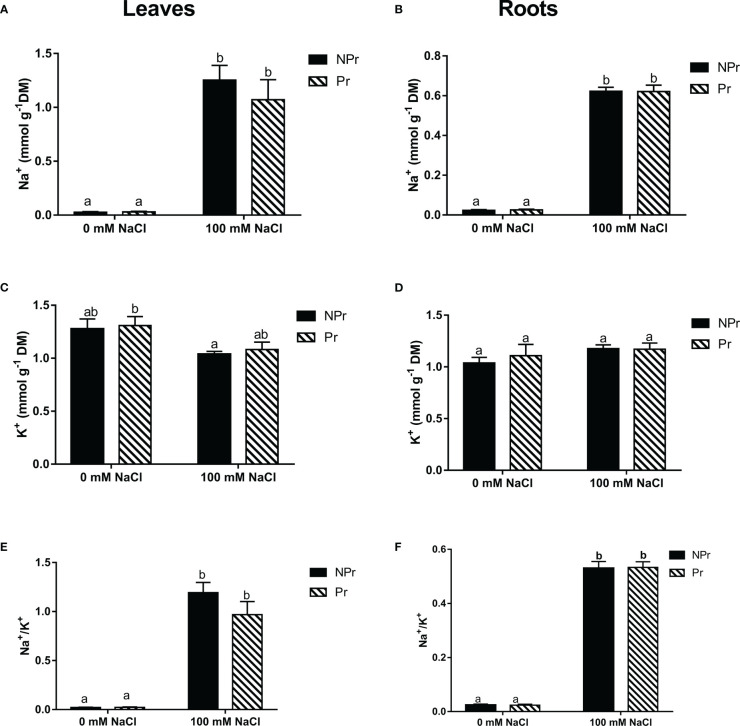
Effects of lettuce seed priming (Pr) with UV-C radiation on Na^+^ and K^+^ contents in leaves **(A, C, E)** and roots **(B, D, F)** at lettuce plants grown in high salinity condition (NaCI 100 mM) or in the absence of NaCI (0 mM). Plants derived from seeds primed are designed by Pr, and non-primed by NPr. Error bars represent the standard errors (n=10). Different letters indicate significant differences at p<0.05 according to Dunn’s test.

High salinity resulted in a heavy accumulation of Na^+^ ions in both leaves and roots (**Figures 5A, B**), and since there was no change in the leaf K^+^ content (**Figures 5C, D** **)**, there was a strong imbalance in the Na^+^/K^+^ ratio as a consequence (**Figures 5E, F** **)**. There were no differences between plants from primed seeds and control plants, regardless of the presence of NaCl in the nutrient solution (**Figure 5**).

### 3.3 Effects of high salinity and UV-C seed priming on chlorophyll content (SPAD values)

**Figure 6:**
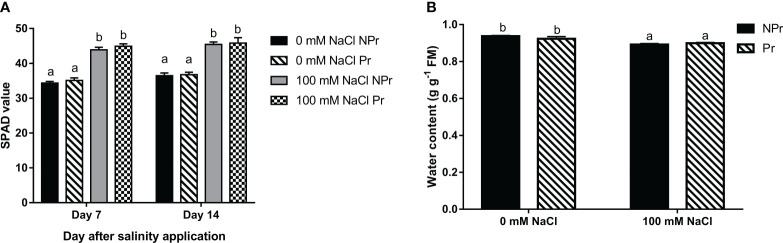
Effects of lettuce seed priming with UV-C radiation in chlorophyll content estimated by SPAD measurements **(A)**, and water content **(B)** of plants grown in high salinity conditions (100 mM NaCI) or in the absence of NaCI (0 mM). Plants derived from seeds primed are designed by Pr, and non-primed by NPr. Error bars represent the standard errors (n=24). Different letters indicate significant differences at p<0.05 according to Dunn’s test.

Plant chlorophyll contents were estimated by SPAD measurements 7 and 14 days after NaCl application. SPAD values were increased by 22% at Day 7 and by 19% at Day 14 in the NaCl-treated plants compared with the control (**Figure 6A**). UV-C priming of seeds did not modify the chlorophyll content of the plants grown under high salinity conditions and did not modify the chlorophyll content of the control plants (**Figure 6A**).

### 3.4 Effects of high salinity and UV-C seed priming on water content

High salinity caused a decrease in leaf tissue hydration status. There were no significant differences between the water content in the leaves of lettuce plants issued from primed seeds (Pr) and the control (NPr) (**Figure 6B**).

### 3.5 Effects of high salinity and UV-C seed priming on plant gas exchange parameters

High salinity resulted in a substantial decrease in A_net_, gs and C_i_ 7 and 14 days after the onset of the NaCl treatment (**Figures 3A–C** **)**. A_net_ was decreased by only 23% in Pr plants vs. 45% in NPr plants at Day 14 (**Figure 3A**). On that day, the photosynthetic electron transport rate (ETR) was also 7% higher in the Pr plants than in the NPr plants in the high salinity treatment (**Figure 3D**).

**Figure 7:**
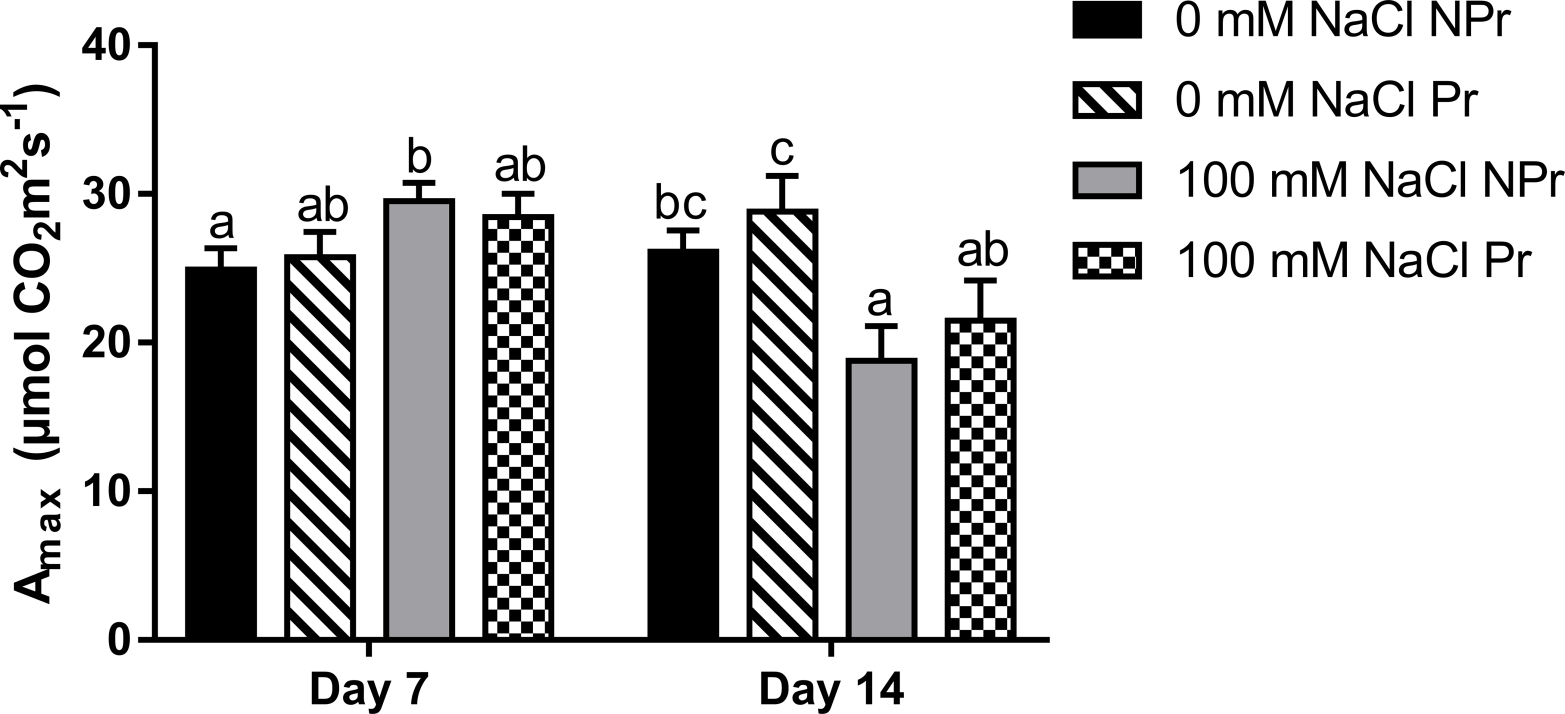
Effects of lettuce seed pnm1ng with UV-C radiation on leaf maximum photosynthesis A_max_ of plants grown in high salinity conditions (100 mM NaCI) or in the absence of NaCI (0 mM). Plants derived from seeds primed are designed by Pr, and non-primed by NPr. Error bars represent the standard errors (n; 10). Different letters Indicate significant differences among means for each mesurment date after salinity Application at p<0.05 according to Dunn’s test.

In NPr plants, high salinity resulted in a 19% increase in A_max_ at Day 7, followed by a 28% decrease (**Figure 7**). UV-C priming of seeds did not impact A_max_ or ETR in either the NaCl treatment or the control, regardless of the date.

**Figure 8:**
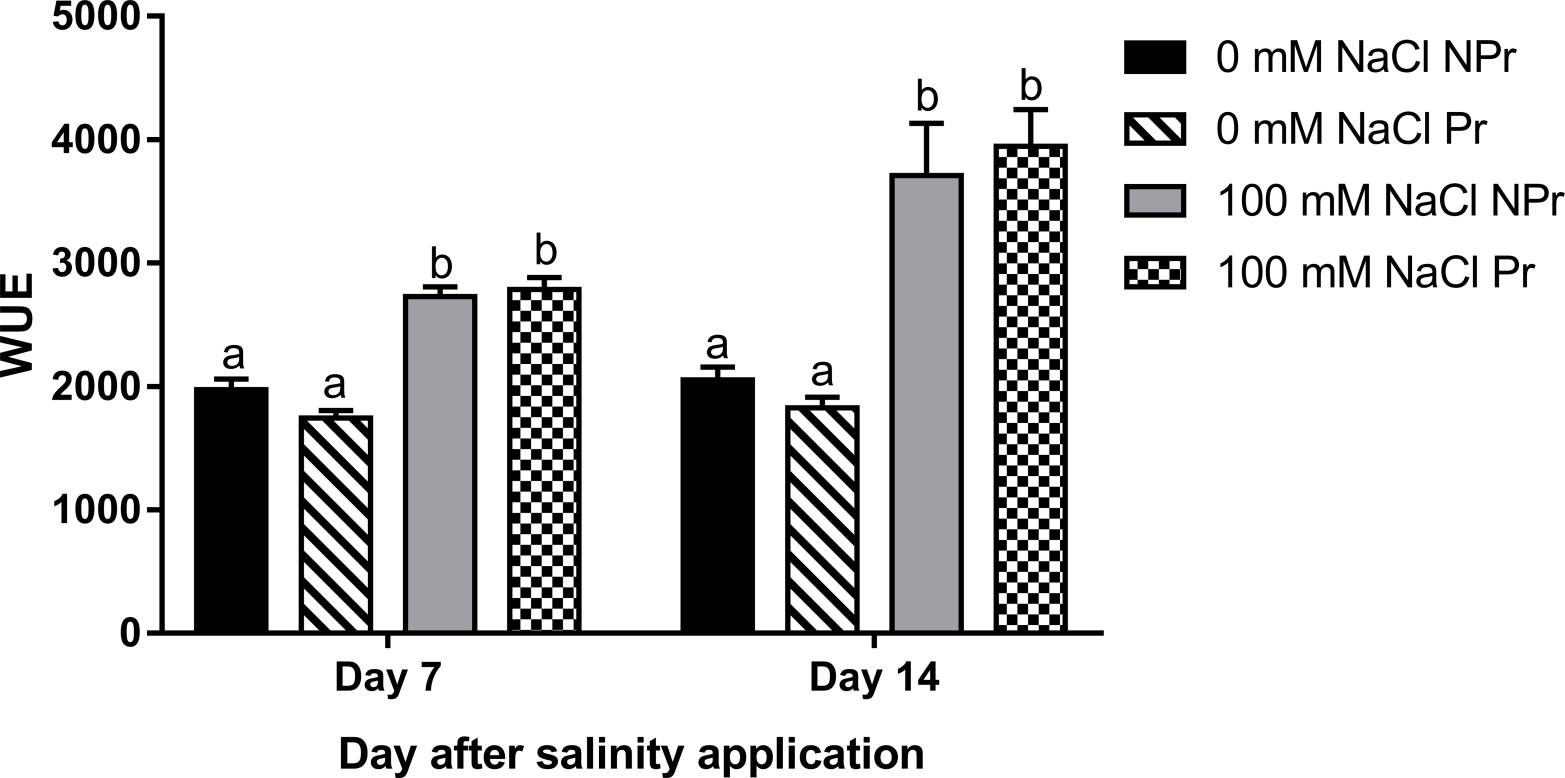
Effects of lettuce seed priming (Pr) with UV-C radiation on water use efficiency (WUE = A_net_/Ei) of plants grown in high salinity conditions (100 mM NaCI) or in the absence of NaCI (0 mM). Plants derived from seeds primed are designed by Pr, and non-primed by NPr. Error bars represent the standard errors (n=10). Different letters indicate significant differences among means for each measurement date, after salinity application at p<0.05 according to Dunn’s test.

Water use efficiency (WUE) was calculated as the ratio of leaf net photosynthesis (A_net_) and leaf transpiration (E). Under high salinity, the WUE was 38% and 81% higher at Day 7 and Day 14, respectively (**Figure 8**). Priming did not modify the WUE at either time.

**Figure 9:**
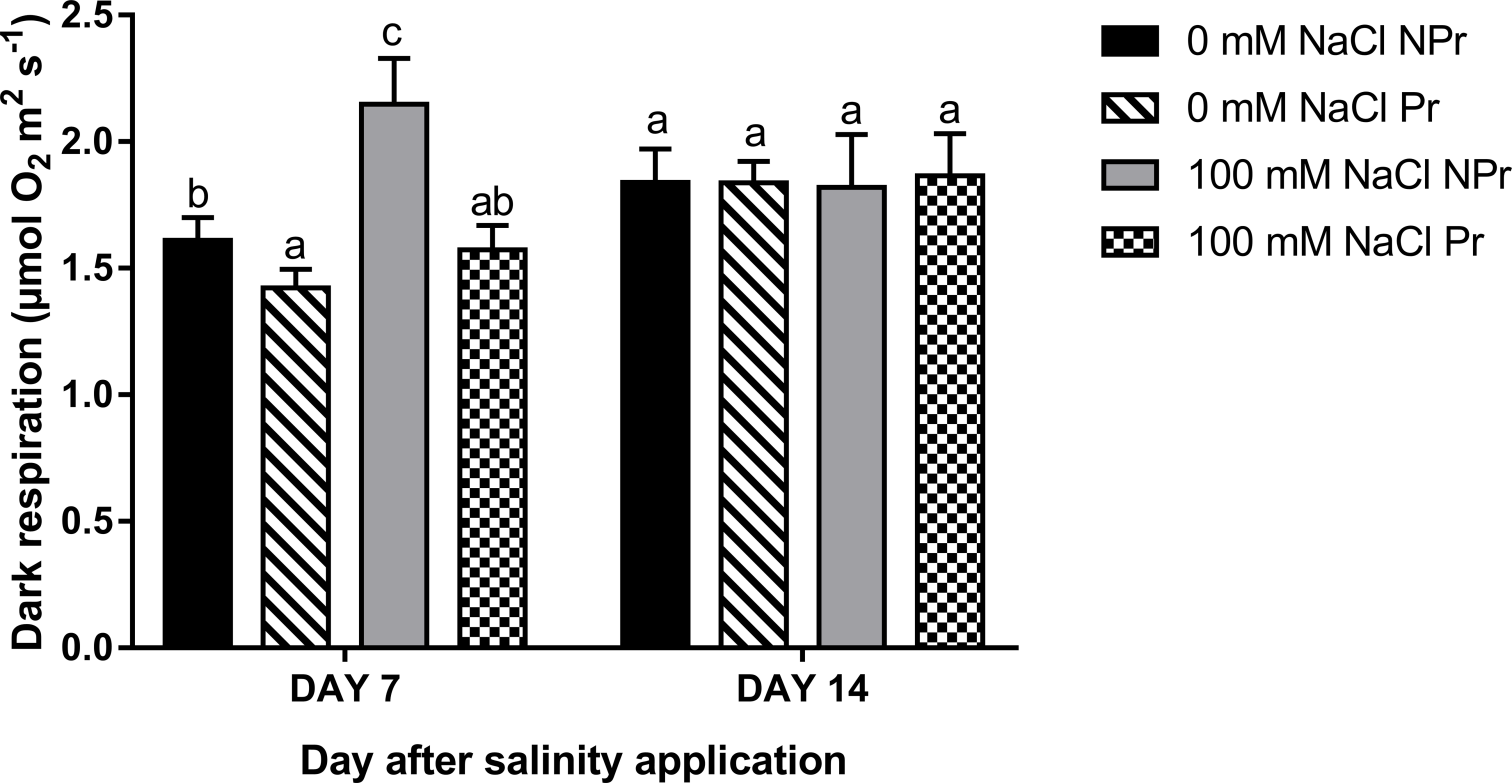
Effects of lettuce seed priming with UV-C radiation on the dark respiration rate (R_d_) of plants grown in high salinity conditions (100 mM NaCI) or in the absence of NaCI (0 mM). Plants derived from seeds primed are designed by Pr, and non-primed by NPr. Error bars represent the standard errors (n=10). Different letters indicate significant differences among means for each mesurement date after salinity application at p<0.05 according to Dunn’s test.

There was an increase in R_d_ in NPr but not Pr plants as a consequence of high salinity at Day 7 (**Figure 9**). A decrease in R_d_ was observed in Pr plants, i.e., as a consequence of UV-C priming of seeds in both NaCl-treated and control plants at Day 7. Such differences were no longer apparent at Day 14 (**Figure 9**).

### 3.6 Effects of high salinity and UV-C seed priming on chlorophyll fluorescence parameters

**Figure 10:**
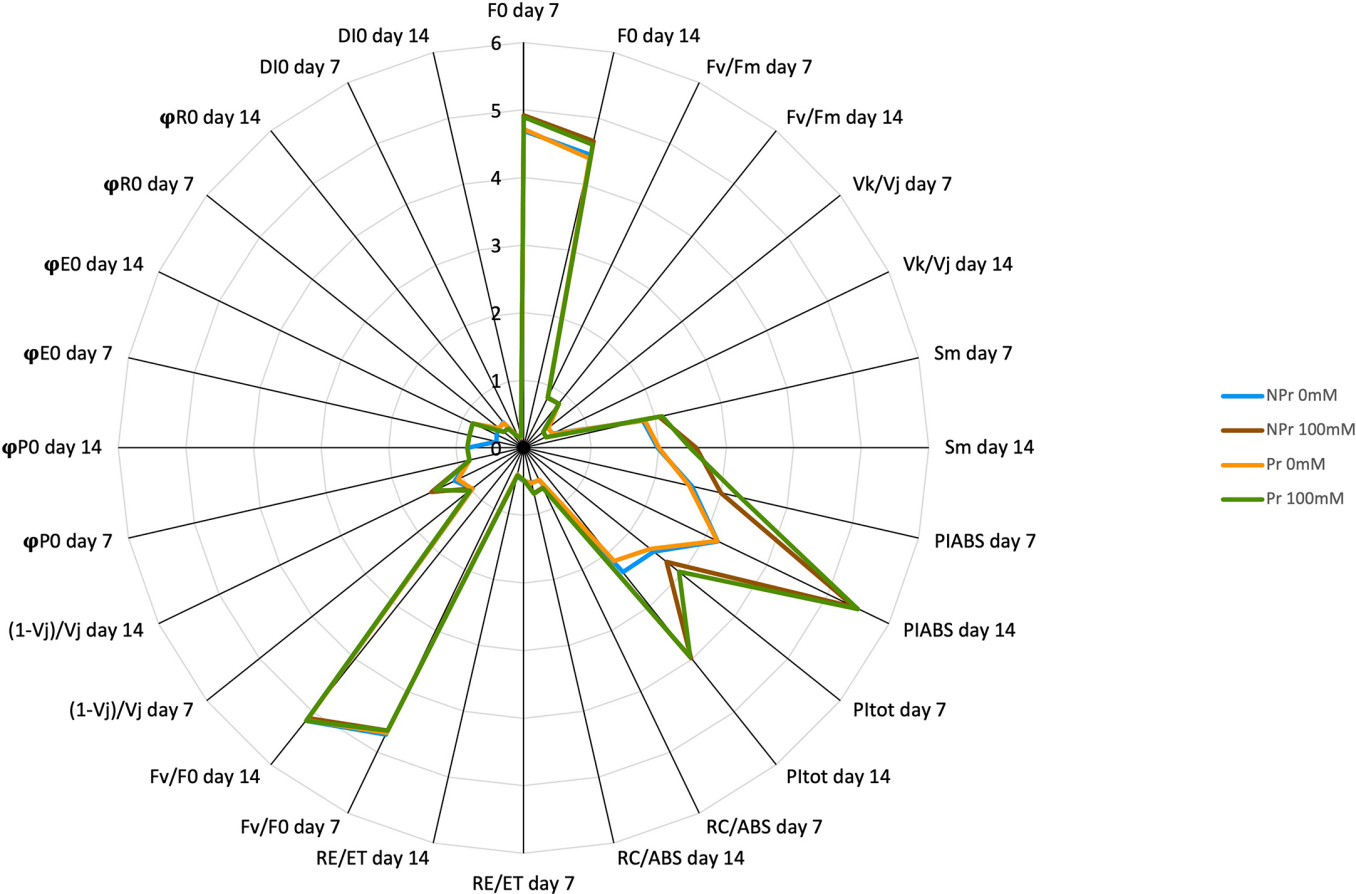
Spider plot of the parameters derived from maximal chlorophyll fluorescence induction curves. See also **Tables 1**–**3**. F0 and Sm values were divided by 1000 and 10, respectively, for the sake of legibility.

The parameters derived from the induction curves of maximal chlorophyll fluorescence, which are believed to be interpretable in terms of damage, are presented in **Table 1** and **Figure 10**. At Days 7 and 14, there was a 5% increase in F_0_ as a consequence of high salinity in both Pr and NPr plants. This increase in F_0_ did not translate into any decrease in F_v_/F_m_. High salinity also resulted in a decrease in V_k_/V_j_ by 19% and 24% at Day 7 and Day 14, respectively. S_m_ was 13 and 29% higher in high salinity-grown plants than in controls at Day 7 and Day 14, respectively.

**Table 1:** Effects of seed priming with UV-C radiation on damage parameters derived from induction curves of maximal chlorophyll fluorescence.

Date of measurement	Salinity stress	F_0_		F_v_/F_m_		V_k_/V_j_		S_m_	
		NPr	Pr	NPr	Pr	NPr	Pr	NPr	Pr
Day 7	0 mM	4689.587^a^	4718.891^a^	0.824^a^	0.823^a^	0.464b	0.468b	18.146^a^	18.372^a^
	100 mM	4922.375^b^	4896.087^b^	0.820^a^	0.822^a^	0.377^a^	0.375^a^	20.576^b^	21.013^b^
Day 14	0 mM	4446.479^a^	4390.109^a^	0.837^a^	0.836^a^	0.480^b^	0.474^b^	19.846^a^	20.016^a^
	100 mM	4655.979^b^	4600.935^b^	0.836^a^	0.837^a^	0.366^a^	0.365^a^	25.551^b^	24.678^b^

Plants from seeds primed are designed by Pr, and non-primed by NPr. Plants grown in high salinity conditions (100 mM NaCl) or in the absence of NaCl (0 mM). F0 is a minimum of fluorescence, Fv/Fm is a maximum quantum yield of primary PSII chemistry, Vk/Vj is an indicator of inactivation of the oxygen evolving complex, and Sm the normalized area above the OJIP curve. Data presented are means of 48 values. Different letters indicate significant differences among means for each measurement date after salinity application at p<0.05 according to Dunn’s test.

Parameters of the quantum yields of electron transport and energy fluxes derived from the induction curves of maximal chlorophyll fluorescence are presented in **Table 2** and **Figure 10**. There was an impact of high salinity on two parameters, namely, φE_0_ and φR_0_. Both parameters were 13% higher as a consequence of high salinity at Day 14. Priming of seeds by UV-C light had no effect and did not modify the responses to high salinity. In addition, high salinity and priming treatments had no impact on φP_0_ or DI_0_/ABS.

**Table 2:** Effects of seed priming (Pr) with UV-C radiation on field parameters derived from induction curves of maximal chlorophyll fluorescence.

Date of measurement	Salinity stress	φP_0_ = (TR_0_/ABS)		φE_0_ = (ET_0_/ABS)		φR_0_ = (RE_0_/ABS)		DI_0_/ABS	
		NPr	Pr	NPr	Pr	NPr	Pr	NPr	Pr
Day 7	0 mM	0.824^a^	0.823^a^	0.410^a^	0.409^a^	0.200^a^	0.198^a^	0.176^a^	0.177^a^
	100mM	0.820^a^	0.822^a^	0.406^a^	0.419^a^	0.192^a^	0.197^a^	0.180^a^	0.178^a^
Day 14	0 mM	0.837^a^	0.836^a^	0.447^a^	0.441^a^	0.188^a^	0.176^a^	0.163^a^	0.164^a^
	100mM	0.836^a^	0.837^a^	0.504^b^	0.499^b^	0.213^b^	0.208^b^	0.164^a^	0.163^a^

Plants from seeds primed are designed by Pr, and non-primed by NPr. Plants grown in high salinity conditions (100 mM NaCl) or in the absence of NaCl (0 mM). TR_0_/ABS maximum trapped exciton flux, ET_0_/ABS Quantum yield of electron transport from QA- to PQ, RE_0_/ABS Quantum yield of electron transport from QA- to final PSI acceptors, and DI_0_/ABS dissipated energy flux per PSII. Data presented are means of 48 values. Different letters indicate significant differences among means for each measurement date after salinity application at p<0.05 according to Dunn’s test.

The results related to the fluorescence parameters contributing to the performance index (PI) are summarized in **Table 3** and **Figure 10**. High salinity resulted in a significant increase in PI_ABS_ values at both Day 7 and Day 14 (+17% at Day 7 and +71% at Day 14 for NPr plants). PI_tot_ was also 69% higher at Day 14 as a consequence of high salinity in the NPr plants. Similarly, higher RC/ABS values were observed as a consequence of high salinity in NPr plants at both dates (+22% at Day 7 and +31% at Day 14). In contrast, F_v_/F_0_ was not affected. (1-V_j_)/V_j_ was 25% lower in NPr plants at Day 14. The UV-C priming treatment did not impact the response to high salinity of these chlorophyll fluorescence parameters.

**Table 3:** Effects of seed priming (Pr) with UV-C radiation on the fluorescence parameters contributing to the performance index (PI).

Date of measurement	Salinity stress	PI_ABS_		PI_tot_		RC/ABS		RE/ET		F_v_/F_0_		(1-V_j_)/V_j_	
		NPr	Pr	NPr	Pr	NPr	Pr	NPr	Pr	NPr	Pr	NPr	Pr
Day 7	0 mM	2.557^a^	2.515^a^	2.464^ab^	2.397^a^	0.535^a^	0.530^a^	0.493^a^	0.487^a^	4.708^a^	4.680^a^	0.971^a^	0.971^a^
	100mM	2.998^b^	3.294^b^	2.707^bc^	2.943^c^	0.655^b^	0.662^b^	0.475^a^	0.472^a^	4.568^a^	4.644^a^	0.963^a^	1.018^a^
Day 14	0 mM	3.190^a^	3.180^a^	2.355^a^	2.144^a^	0.526^a^	0.534^a^	0.424^a^	0.404^a^	5.167^a^	5.156^a^	1.132^b^	1.077^b^
	100mM	5.439^b^	5.493^b^	3.978^b^	3.950^b^	0.688^b^	0.695^b^	0.421^a^	0.417^a^	5.114^a^	5.167^a^	1.508^a^	1.451^a^

Plants from seeds primed are designed by Pr, and non-primed by NPr. Plants grown in high salinity conditions (100 mM NaCl) or in the absence of NaCl (0 mM). PI_ABS_ the performance index for energy conservation from photons absorbed by PSII to the reduction of intersystem electron acceptors, PI_tot_ the performance index for energy conservation from photons absorbed by PSII antenna until the reduction of PSI acceptors, RC/ABS the density of active PSII reaction centers expressed on the base of the quantity of light absorbed by the antenna, RE/ET the electron transport flux from Q_B_ to PSI acceptors, F_v_/F_0_ the contribution to the PI of the light reactions for primary photochemistry, and (1-V_j_)/V_j_ the performance due to the conversion of excitation energy to photosynthetic electron transport, and Data presented are means of 48 values. Different letters indicate significant differences among means for each measurment date after salinity application at p<0.05 according to Dunn’s test.

### 3.7 Effects of high salinity and UV-C seed priming on phytohormones

**Figure 11:**
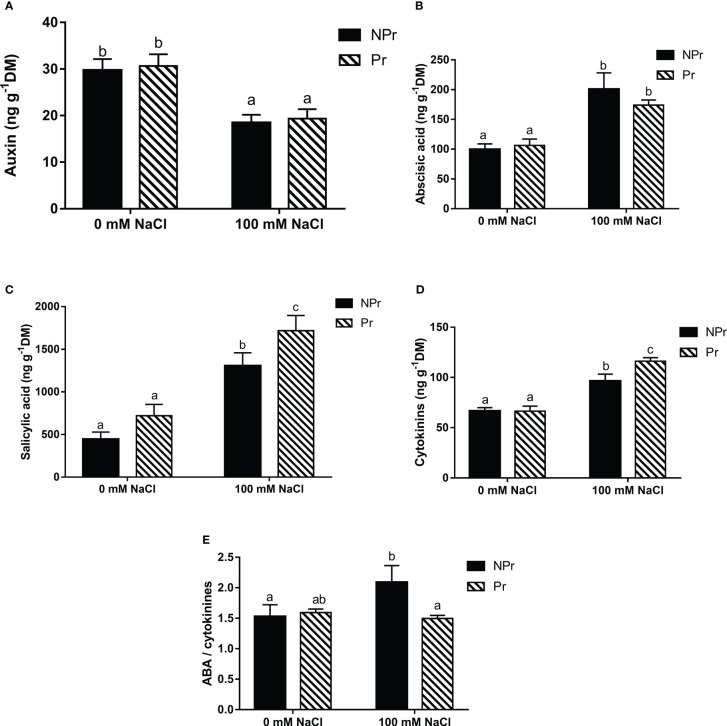
Effects of lettuce seed priming (Pr) with UV-C radiatoi n on phytohormones endogenous concentrations of plants grown in high salinity conditions (100 mM NaCI). auxin **(A)**, abscisic acid (ABA) **(B)**, sailcylic acid **(C)**, cytokinins **(D)**, and ABA/cytokinins ratio **(E)**. Error bars represent the standard errors (n=6). Different letters indicate significant dfiferences among means for each mesurment date after salinity application at p<0.05 according to Dunn’s test.

High salinity caused many changes in the phytohormonal status of lettuce leaves. In NPr plants, it resulted in a 38% decrease in auxin content (**Figure 11A**). For other hormones, an increase of 101% of ABA (**Figure 11B**), 194% of SA (**Figure 11C**) and 44% of CKs (**Figure 11D**) were obtained at 100 mM NaCl. The ABA/CKs ratio (**Figure 11E**) also increased by 37% in NPr plants.

Seed priming by UV-C light maintained the levels of ABA and auxin compared to NPr plants under NaCl conditions (**Figures 11A, B**). Seed priming induced a 31% increase in the SA concentration (**Figure 11C**) and a 21% increase in the CKs concentration (**Figure 11D**) compared to NPr plants under salinity conditions. The ratio of ABA to CKs remained unchanged in plants grown in the absence of NaCl (**Figure 11E**).

## 4 Discussion

### 4.1 Features of salinity stress in our trial

High salinity is known to impact plant photosynthesis and growth negatively, primarily through osmotic stress and then salt toxicity (Munns, 2002). Tolerance to high salinity is generally due to the selectivity of K^+^ absorption to maintain Na^+^/K^+^ homeostasis in salt stress conditions (Tester and Davenport, 2003; Gupta and Huang, 2014). In our trial, we found, as expected, a substantial increase in Na^+^ content in leaves and roots that was however not associated to a decrease in either leaf or root K^+^ content of the lettuce plants submitted to high salinity. There are important differences with the observations of Ouhibi et al. (2014). Firstly, Na^+^ leaf content reached only 1.2 mmol g^-1^ DM in our trial (**Figure 5A**) vs. 2.0 mmol g^-1^ DM in their trial. The difference was even more marked for Na^+^ root content (**Figure 5B**). Moreover, K^+^ leaf and root contents were not decreased as a consequence of high salinity in our trial (**Figures 5C, D**), by contrast to the observations of Ouhibi et al. (2014).

These differences are surprising in the first place since the same concentration of 100 mM NaCl was applied in both trials. But then, plants were grown in pots, in a conventional way by Ouhibi et al. (2014), whereas plants in our trial were grown in a full hydroponic system, where roots developed in a nutrient solution constantly oxygenated by bubbling. In such a system, ion convection is high because of bubbling, which prevents very effectively the formation of ion gradients in the root zone. Such gradients form naturally for all ions that are in excess in the supply relative to the uptake by plants. They form in traditional soilless systems based on the use of growing media, because the latter have no or little cation exchange capacity and because ion diffusion is too slow a process to homogenize ion concentrations across the nutrient solution (Urban and Urban, 2010). In commercial greenhouses equipped for soilless culture in growing media, ion gradients are routinely reduced by the supply of nutrient solution in excess, which leads to the well-known practice of “drainage” (Urban and Urban, 2010). Interestingly, Cabrera and Perdomo (2003) challenged the classification of rose plants as salinity-sensitive on the basis of observations they made in soilless systems with drainage. We formulate the hypothesis that we maintained 100 mM in the root zone of lettuce plants in our trial thanks to bubbling, as was our intention, whereas the NaCl concentration increased uncontrolled in the trial of Ouhibi et al. (2014) in the absence of a strategy of irrigation with drainage. In other words, the differences in Na^+^ and K^+^ contents between both trials reflect differences in intensity of salinity stress. Even though Na^+^ contents increased in both leaves and roots as a consequence of high salinity in our trial, K^+^ homeostasis suggests that K^+^ transport systems were not mobilized (Lu et al., 2020).

Consistent with the idea that the salt toxicity component of salt stress was moderate or even inexistent in our trial is the absence of visual symptoms of damage. Our ChlF data moreover suggest that stimulation of photorespiration, possibly in addition to other alternative routes for electrons in excess and ROS scavenging processes, proved efficient in preventing damage to the photosynthetic machinery. The hypothesis that photorespiration was increased as a consequence of high salinity in our trial, is substantiated by the fact that the photosynthetic electron rate (ETR) was maintained while A_net_ decreased on both Days 7 and 14 (**Figures 3A, D**). Concerning ChlF data, on both Day 7 and Day 14, we found a moderate increase in F_0_, which did not result in a decrease in F_v_/F_m_, whereas V_k_/V_j_ was found to decrease and S_m_ to increase under conditions of high salinity (**Table 1**). According to Ripoll et al. (2016) and Urban et al. (2017), such changes do not support the hypothesis of a damaging effect on the photomachinery (Ripoll et al., 2016), and they therefore confirm that salt stress was moderate in this trial.

### 4.2 Hormonal changes associated with high salinity

**Figure 12:**
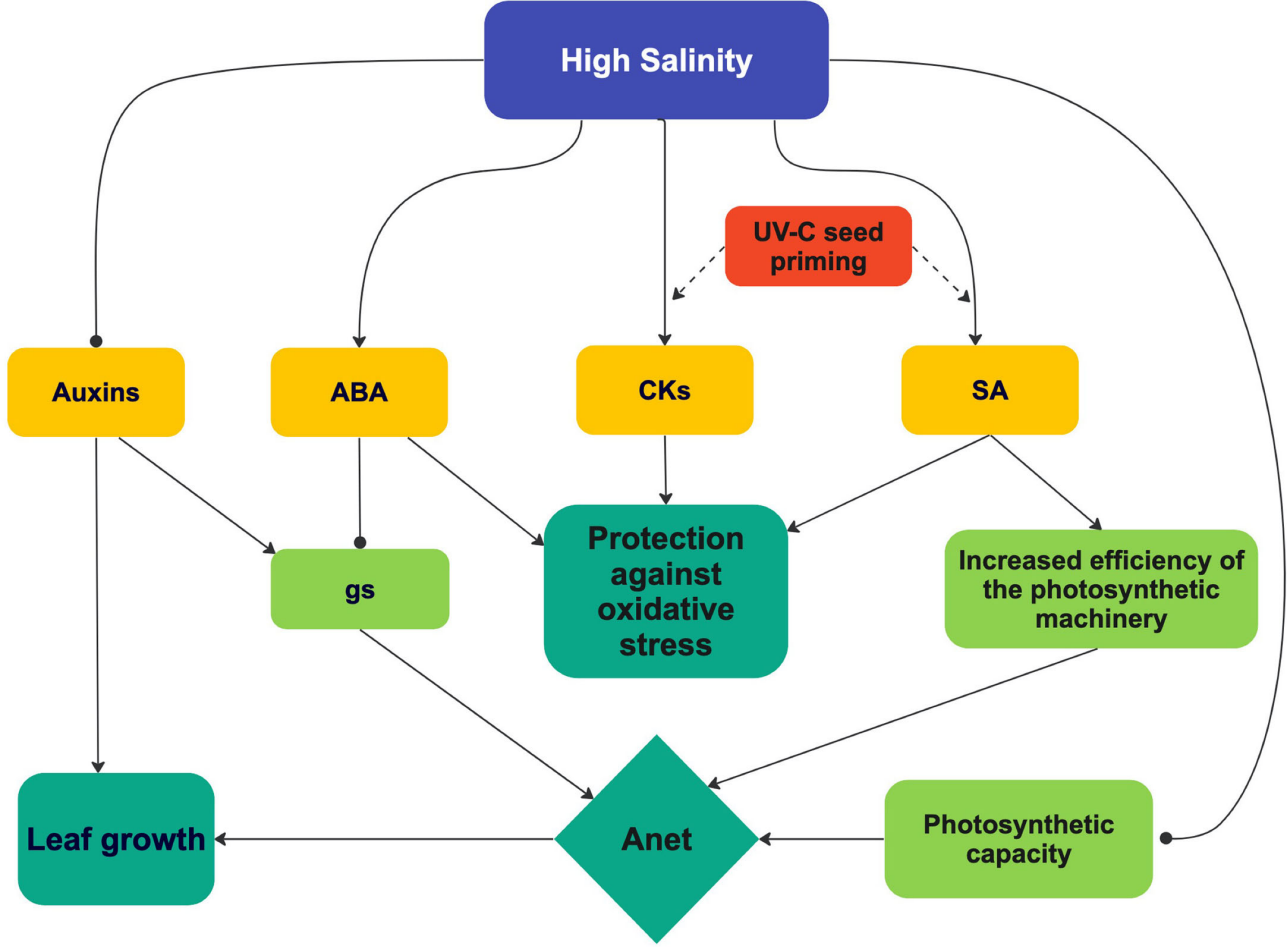
A tentative and integrated view of the effect of high salinity and UV-C priming on hormone concentrations, photosynthesis and leaf growth. See text for details. UV-C priming resulted in a reinforced effect of high salinity on SA and CKs concentrations.

Protection of the components of the photosynthetic electron transfer chain plays an important role in the adaptative response of plants to high salinity (Evelin et al., 2019). Such protective mechanisms may be attributed to SA, which was found to be dramatically increased in leaves of plants grown at 100 mM NaCl compared to control leaves (**Figure 11C**). One major hypothesis is that SA exerts a protective effect against stress by stimulating antioxidant responses (**Figure 12**). There is indeed a wealth of scientific evidence that SA stimulates the activity of antioxidant enzymes (superoxide dismutase, peroxidases, catalase) or enzymes of antioxidant systems, such as glutathion reductase. See, for instance, Dong et al. (2014) on this topic. Consistent with the idea that SA increase exerted a protective effect on the photosynthetic machinery and its functioning in plants grown at high salinity is the maintenance of nearly all ChlF parameters at the same levels than in the control (**Tables 1**–**3**). There was even an increase in S_m_ and a decrease in V_k_/V_j_ on both Days 7 and 14 (**Table 1**), and an increase in φE_0_ and φR_0_ at Day 14 (**Table 2**), and PI, the latter attributable to increases in RC/ABS and (1-V_j_)/V_j_ at Day 14 (**Table 3**). These changes indicate not only that the photosynthetic machinery was not damaged but that its functioning was improved. Our data more specifically suggest that quantum efficiency of electron transport from Q_A_ to PQ and to PSI acceptors was improved. Our observations confirm the ones of Lotfi et al. (2020) who also found a bettering of ChlF parameters, attested notably by an increase in the area derived from analysis of maximal ChlF induction curves, in *Vicia radiata* plants treated with SA and grown at high salinity.

The high levels of SA in leaves of lettuce plants grown at 100 mM NaCl were associated with an increase in leaf ABA and cytokinins, and a decrease in auxin concentrations (**Figure 11**), which confirms a majority of observations about the effects of high salinity on hormone concentrations in plants and also about interactions between hormones in plant tolerance against stress.

The high ABA endogenous concentration in leaves of lettuce plants grown at 100 mM NaCl (**Figure 11B**) is probably associated with the substantial decrease in g_s_ we observed in this trial (**Figure 3B**) (Jones et al., 1980; Cowan, 1982; Davies and Zhang, 1991). It may also have played a role in homeostasis or even improvement of ChlF fluorescence data, considering the role of ABA in antioxidant activities (Zhang et al., 2006; Chaves et al., 2009). It may not be excluded that high SA reinforced ABA. Szepesi et al. (2009) found indeed that exogeneous applications of SA increased ABA accumulation and improved stress tolerance on tomato plants grown under high salinity conditions.

Auxins are known to play a key role in growth and development in plants, notably root differentiation (Bali et al., 2017). We observed that reduced growth of the aerial part under high salinity conditions (**Figure 4**) was associated with a decrease in leaf auxin endogenous concentration (**Figure 11A**), which is consistent with previous observations in other species such as maize (Ribaut and Pilet, 1991; Ribaut and Pilet, 1994) and tomato (Dunlap, 1996). Interestingly, salinity stress resulted in a strong reduction of root growth in the trial of Ouhibi et al. (2014), but not in our trial (**Figure 4F**). We may attribute this difference to the fact that auxin depletion was relatively moderate in our trial, i.e. sufficient to limit leaf but not root development, another argument in favor of the idea that salinity stress was moderate. ABA and auxin pathways interact antagonistically in plant tolerance mechanisms. Auxin can promote stomatal opening and reverse stomatal closure induced by ABA (Levitt et al., 1987). This clearly did not happen for plants grown at high salinity in this trial (**Figure 3B**), arguably because high SA interfered with auxin signaling in an antagonist way (Iglesias et al., 2011). MicroRNA miR393, the main regulator of auxin receptors TIR1, AFB2 and AFB, was shown to be stimulated under salinity stress (Sunkar et al., 2007). Navarro et al. (2006) found that stress tolerance was associated with miR393-triggered repression of auxin receptors. Observations by (Iglesias et al., 2011) on Arabidopsis mutants have shown that tolerance against salinity stress increases in double tir1 and afb2 mutants, whereas SA increases PR1 genes in these mutants. Such a mechanism is part of an integrated mechanism allowing plants to deal with multiple stresses (Agtuca et al., 2014).

Leaf endogenous concentration of CKs were higher in plants grown at high salinity than in the control (**Figure 11D**), which is consistent with observations in such species as *Arabidopsis thaliana* (Prerostova et al., 2017), apple (Keshishian et al., 2018), rice (Joshi et al., 2018) or tomato (Feng et al., 2019), but stays in contrasts with others like wheat (Avalbaev et al., 2016) or maize (Zhang et al., 2018). In fact, the way CKs are associated with tolerance to high salinity does not seem to result only from the specific responses of the different species but also to duration of stress and the way hormone signaling systems interact in the presence of stress (Mandal et al., 2022). Within this view, we may hypothesize that the decrease in the CKs/ABA ratio in the plants grown at high salinity (**Figure 11E**) is attributable to downregulation of CKs production by stress-induced ABA accumulation (Li et al., 2016). Even if CKs synthesis was downregulated, leaf CKs concentrations increased all the same in the plants grown at high salinity. This may have contributed to the protection of the photosynthetic machinery (Cortleven et al., 2014; Cortleven and Schmülling, 2015), as the near-homeostasis of ChlF parameters would suggest (**Tables 1**–**3**).

### 4.3 Stomatal and non-stomatal limitations of photosynthesis in high salinity conditions-consequences on growth parameters

There was a strong decrease in A_net_ at Days 7 and 14 (**Figure 3A**). This decrease is consistent with similar observations made on lettuce plants grown under conditions of high salinity (De Pascale and Barbieri, 1995; Ekinci et al., 2012; Huang et al., 2019; Shin et al., 2020). The high salinity-associated decrease in A_net_ may be attributed to a decrease in g_s_ and C_i_ at Days 7 and 14 (**Figures 3B, C** **)**, and moreover to an increase in mitochondrial respiration at Day 7 (**Figure 9**) and a decrease in photosynthetic capacity (A_max_) at Day 14 (**Figure 7**).

The decrease of g_s_ in the leaves of lettuce plants grown at high salinity may be attributable to the increase in ABA content (**Figure 11B**). ABA signaling from roots to stomata is sensed by guard cells responsible for stomatal closure (Karimi et al., 2021). This phenomenon is very important to limit water losses through leaf transpiration (Niu et al., 2018). Our results showed a decrease in g_s_ and an increase in photosynthetic water-use efficiency (WUE = A_net_/E), with E representing transpiration. Increases in WUE under high salinity conditions have been reported in many studies (Ashraf, 2001; Omamt et al., 2006) and they represent a normal response to moderately reduced g_s_ levels (Damour et al., 2010).

Photosynthetic capacity may be related to the chlorophyll content expressed on a leaf area basis, which determines the maximal rate of photosynthetic electron transport (Lawlor and Tezara, 2009). Our SPAD data do not support the view that the chlorophyll content was decreased by high salinity conditions (**Figure 6A**). In contrast, there was an increase in SPAD values, consistent with previous studies about the stimulating effect of high salinity on leaf chlorophyll content (Ashraf, 2004; Ashraf et al., 2008; Ekinci et al., 2012). Higher SPAD values are not attributable to a decrease in leaf water content in our trial, since the latter decreased only moderately as consequence of high salinity (**Figure 6B**), and therefore may rather reflect plant adaptation through modifications of cell and tissue anatomy that result in a higher chloroplast density per unit leaf area (Acosta-Motos et al., 2017; Adhikari et al., 2019). At Day 7, the higher SPAD values were correlated with higher A_max_ values in the leaves of the plants grown at salinity (**Figures 6A**, **7**); but at day 14, A_max_ was lower, suggesting that factors other than leaf chlorophyll content were responsible of downregulation of photosynthetic capacity. The fact that A_max_ was lower at Day 14 only, suggests that high salinity-induced non stomatal limitations of photosynthesis may take some time before expressing themselves (Brugnoli and Björkman, 1992; Sarabi et al., 2019; Pan et al., 2021).

An increase in mitochondrial respiration, as the one we observed on Day 7 (**Figure 9**), is a common feature of adaptation to high salinity, even though there are contradictory observations (Jacoby et al., 2011). On Day 14, this increase was no more visible in our trial. Interpretation is not easy because the role of respiration in plant adaptation is a complex one, but it can at least be said that it is a negative feature when considering leaf A_net_, the plant carbon budget and the resulting crop performance (Jacoby et al., 2011). On Day 7, we can state than higher respiration contributed to lower A_net_ in plants grown at high salinity.

The decrease in A_net_ we observed in plants grown in high salinity conditions accounts for the decreases in growth parameters (**Figure 4**). The latter are consistent with observations made on lettuce (Barassi et al., 2006; Ünlükara et al., 2008; Al-Maskri et al., 2010; Ekinci et al., 2012; Turhan et al., 2014; Ahmed et al., 2019), and other plant species, such as sorghum, rice and tomato (Gill et al., 2001; Minh et al., 2016; Rodríguez-Ortega et al., 2019) under high salinity conditions.

### 4.4 Effect of seed priming by UV-C light

The mitigating effect of UV-C seed priming on plant growth and yield in conditions of high salinity (**Figures 4A–D**) on Day 14 is consistent with other observations about the positive effect of UV-C seed priming. Brown et al. (2001) observed that UV-C light at 3.6 kJ m^-2^ results in a larger cabbage head diameter. A similar positive effect was observed in groundnut and mungo bean for seeds submitted to 30 minutes of UV-C radiation (Siddiqui et al., 2011). Compared to the control, mungo bean plants from Pr seeds exhibited leaves with a higher fresh mass (+100%) and leaf area (+40%). The same trend was observed in groundnut plants: +20% and +85% for the fresh mass and leaf area, respectively (Siddiqui et al., 2011).

Our observations confirm partly the ones of Ouhibi et al. (2014). For example, that study showed a sizable increase in the leaf area of plants issued from primed seeds compared to control plants at 0.85 kJ m^-2^. This enhancement, evaluated at +50%, was very close to our observations (+48%). However, the mitigating effect of priming by UV-C light on growth of lettuce plants at high salinity was globally less pronounced in our trial than in theirs. We attribute this difference to the fact that we controlled NaCl concentration in the root zone of plants with our bubbling system, as exposed above.

The positive effect of UV-C seed priming on growth parameters of plants at high salinity can be explained by its effect on A_net_ (**Figure 12**), though this effect was statistically significant only at Day 14 (**Figure 3A**). The increase in A_net_ at Day 14 in our study was associated with an increase in ETR (**Figure 3D**) but not in SPAD values, gs, C_i_ or A_max_ (**Figures 6**, **3B, C**, **7A**), nor with a decrease in R_d_ (**Figure 8**). ETR data notably suggest that priming seeds by UV-C light improves the efficiency of light use or photochemical quenching, which both have been suggested as important tolerance mechanisms in plants submitted to high salinity (Pan et al., 2021).

The positive effect of seed priming by UV-C light on photosynthesis may be attributed to the effect of priming on SA and CKs leaf concentrations (**Figures 11C, E**) and the roles played by them on photosynthesis (**Figure 12**). There is evidence notably that SA stimulates Rubisco activity (Janda et al., 2014; Poór et al., 2019), whereas CKs increase the expression of photosynthetic genes encoding proteins of PSI, PSII, and the cytochrome b6f (Cytb6f) complex, which are involved in the photosynthetic electron transport chain (Rivero et al., 2007; Rivero et al., 2009; Rivero et al., 2010). Such a positive effect of CKs on the photosynthetic electron transport chain could translate into a positive effect on ETR. Indeed, this what was observable in plants from primed seeds grown at high salinity, at Days 7 and 14 (**Figure 3D**). Interestingly, priming resulted in the maintenance of the CKs/ABA ratio at Day 14 in the plants grown at high salinity (**Figure 11E**), which supports the idea that stress was less marked in plants from primed seeds and therefore that priming reduced the negative effect of high salinity.

The fact that leaf SA and CKs concentrations were higher in growing plants from UV-C-treated non-germinated seeds is intriguing and raises questions about UV-C light perception by seed coat and also about the mechanisms behind priming that lead to such increases. Considering that UV-C light can be at the origin of epigenetic responses (Molinier, 2017; de Oliveira et al., 2020), it would be interesting to analyze specific molecular markers, such as the ones associated with histone methylation (Müller-Xing et al., 2014).

## 5 Conclusion

Our observations were made under the conditions of strict control of NaCl concentration in the nutrient solution. Under such conditions, salinity stress may be considered as moderate and probably involves only osmotic stress and not toxicity due to excess Na^+^ accumulation. We hypothesize that this may account for the less marked mitigating effect of seed priming by UV-C light on growth parameters we found in our trial compared to the one of Ouhibi et al. (2014). As a practical consequence, seed priming by UV-C light appears more recommendable for conditions of severe than moderate salinity stress. The dynamic of ions is very different in soil than in soilless conditions. Transpiration rates also are different in field than in controlled conditions. High transpiration may notably exacerbate the negative effect of high salinity in sandy soils with low cationic exchange capacity. We therefore recommend field trials to be conducted across a broad range of climatic and soil conditions with the objective of assessing the agronomic benefits of seed priming by UV-C light.

SA and cytokinins accumulation probably played an important role in the positive effects exerted by seed priming on photosynthesis and growth of lettuce plants grown in conditions of high salinity (**Figure 12**). The pivotal role played by SA in conjunction with other hormones in tolerance mechanisms against abiotic stress, as it emerges in the current literature, represents a strong incentive for investigating the mechanisms that lead SA leaf concentration to increase in lettuce plants from UV-C-treated non-germinated seeds. Besides the question of UV-C light perception by non-germinated seeds, the epigenetic hypothesis behind priming certainly deserves to be studied.

## Data availability statement

The raw data supporting the conclusions of this article will be made available by the authors, without undue reservation.

## Author contributions

SF, conceptualization, methodology, software, validation, formal analysis, investigation, resources, data curation, writing - original draft, writing - review and editing, and visualization. JA, conceptualization, methodology, software, validation, formal analysis, investigation, resources, data curation, writing - original draft, writing - review & editing, visualization, supervision, project administration, and funding acquisition. FL-L, methodology, validation, writing - review and editing, and visualization. YL, methodology and data curation. FP, conceptualization, resources, and funding acquisition. LU, conceptualization, methodology, software, validation, formal analysis, investigation, resources, data curation, writing - original draft, writing - review & editing, visualization, supervision, project administration, and funding acquisition. All authors contributed to the article and approved the submitted version.
